# Partial Substitution of Soybean Meal with a Yeast-Fermented Vinasse Protein Source: Effects on Milk Yield, Composition, and Serum Biochemistry in Crossbred Dairy Cows

**DOI:** 10.3390/metabo16080518

**Published:** 2026-07-23

**Authors:** Ahmet Akdag

**Affiliations:** Department of Animal Science, Faculty of Agriculture, Eskisehir Osmangazi University, Eskisehir 26480, Türkiye; ahmeta@ogu.edu.tr

**Keywords:** animal nutrition, dairy, feed ingredients, metabolic profiling

## Abstract

Background/Objectives: This study aimed to compare the effects of two distinct dietary protein sources, soybean meal (SBM, 454 g CP/kg dry matter [DM]) and fermented protein source (FPS, 635 g CP/kg DM), on milk yield (MY), milk quality traits, and serum biochemistry in crossbred dairy cows. Methods: This study employed a randomized complete block design with four groups (*n* = 6 per group): Control (120 g/kg SBM), FPS30 (30 g/kg FPS and 75 g/kg SBM), FPS40 (40 g/kg FPS and 60 g/kg SBM), and FPS50 (50 g/kg FPS and 45 g/kg SBM). Results: After 66 days (21-day adaptation period and 45-day feeding trial), DM intake, milk protein content, and milk density did not differ significantly across groups. However, MYs were significantly higher in the FPS50 group (*p* = 0.043). Additionally, milk fat (*p* = 0.021), DM (*p* = 0.013), solids-non-fat (SNF, *p* = 0.047), and lactose content (*p* = 0.007) were significantly higher in the FPS groups. Although serum biochemistry did not differ across groups on day 0, blood urea nitrogen (BUN, *p* = 0.044) and serum Ca (*p* = 0.041), P (*p* = 0.039), and Mg (*p* = 0.041) concentrations were significantly lower in the FPS groups on day 66. Moreover, FPS intake correlated positively with MY (*R* = 0.680), milk DM (*R* = 0.706), fat (*R* = 0.665), SNF (*R* = 0.566), lactose content (*R* = 0.653), and fat-to-protein ratio (*R* = 0.507) and negatively with milk density (*R* = −0.429) and mineral content (*R* = −0.271). Conclusions: These findings indicate that FPS is effective in increasing productivity and improving/maintaining milk quality traits in cows fed diets substituting specific amounts of SBM with FPS. The induced reduction in BUN can be interpreted as increased protein utilization.

## 1. Introduction

The livestock feed industry is confronted with a persistent global challenge concerning the availability of feed resources due to a rise in the cost of conventional animal feed ingredients [1]. Increasing competition for land, particularly for industrialization, is intensifying competition between feed production for monogastric and ruminant animals and grain production for human consumption, thereby increasing production costs and constraining commercial operations for farmers reliant on traditional cereals and meal sources in animal diets [2,3]. Consequently, it is inevitable to investigate alternative feed resources that are less expensive, locally sourced, and unconventional [4].

Currently, soybean meal (SBM) is the most common protein-rich feedstuff in the livestock sector for meat and milk production [5] due to its high proportion of rumen-digestible protein (RDP). It is a by-product of soybean oil extraction and accounts for approximately 70% of the oilseed meal consumed globally [6]. However, due to the limited capacity and comparatively higher production costs of SBM, animal nutritionists are exploring alternative dietary protein sources [7].

Fermentation has been identified as a viable method for extracting bioactive components due to microorganisms’ ability to hydrolyze the complex carbohydrates and proteins present in plant tissues, thereby liberating free phenolic compounds, proteins, peptides, and amino acids [8]. Therefore, fermentation is considered a highly effective method for enhancing the nutritional value of agricultural by-products. It primarily involves the degradation of fiber (cellulose and lignin) and the elimination of anti-nutritional factors, thereby enhancing the bioavailability of nutrients and promoting health benefits [9,10]. The benefits of fermentation include enhanced vitamin content and enzyme activity, as well as the synthesis of growth-promoting and anti-pathogen substances [11,12]. Therefore, many studies have examined ways to increase the amount of free phenolic and protein content in fermented products, produce bioactive peptides, and eliminate anti-nutritional factors [13,14,15].

By-products of the yeast industry are generally known as yeast culture and vinasse. Yeast culture primarily consists of yeast cell metabolites, fermentation medium components, and inactive yeast cells [16,17]. Vinasse primarily consists of crude protein (CP, particularly aspartic acid, glutamic acid, and betaine) and ash (containing particularly potassium [K]) formed during molasses fermentation [18]. Chuppa-Tostain et al. [19] reported that vinasse contains inactive *Saccharomyces cerevisiae*, which can ameliorate the rumen environment and promote the growth of rumen microorganisms. Fermented feed can also contain wheat bran, a by-product of the milling industry with a high fiber content and an average CP content, as it is relatively easy to obtain and inexpensive for animal nutrition.

Although numerous studies have investigated alternative protein sources to SBM in dairy nutrition, a scientific gap remains regarding the fermentation of a vinasse-based product, its comparison with SBM, and its effects on the blood biochemistry of dairy cattle. Therefore, this study aimed to investigate the effects of substituting specific amounts of SBM in the diets of crossbred dairy cattle, fed isocaloric and isonitrogenous diets, with an FPS (*Aspergillus oryzae*- and *Bacillus subtilis*-based) containing high non-protein nitrogen (NPN) content, inactive yeast, yeast cell metabolites, and bioactive compounds on milk yield (MY), milk composition, and blood biochemistry. It was hypothesized that supplementing SBM with FPS would improve dairy cows’ performance, milk components, and blood biochemistry.

## 2. Materials and Methods

### 2.1. Animals and Diets

This study was conducted on a commercial farm in the Tasova District, Amasya Province, Türkiye, located at an altitude of 764 m at coordinates 40°40′48″ N and 35°52′47″ E. It was approved by the Local Ethics Committee for Animal Experiments of Ondokuz Mayıs University (Protocol No: 2024/4), and the cows were managed, cared for, and fed in accordance with its guidelines.

This study employed a randomized complete block design with four groups (*n* = 6 per group), with each cow considered an experimental unit. Cows were housed, fed and randomized individually. The animals were first blocked by parity (2nd, 3rd and 4th) and then randomly allocated to one of four treatment groups while maintaining a balanced distribution for days in milk (DIM) across treatments at the start of the study. In this study, pedigree-registered Holstein × Simmental crossbreed cows with a mean body weight (BW) of 478 ± 19.4 kg and initial MY (IMY) of 11.60 ± 0.94 kg were employed. Cows were selected for this study based on body condition scores (BCS), DIM, parity, MY, and BW to ensure homogeneity across groups. Regarding BW, cows weighing 450–500 kg were selected for this study. Regarding BCS, all cows included in this study had a BCS of 3.00, determined as described by Ferguson et al. [20]. Regarding DIM, four of the six cows in each group were in the first 70 days of lactation, while two were between 70 and 100 days. Regarding parity, three of the six cows were in the second lactation, two were in the third, and one was in the fourth. Regarding MY, cows’ MY records were carefully reviewed to ensure that each group comprised cows with analogous MYs for each lactation and parity. Moreover, the sample size was determined based on practical considerations, including the availability of suitable animals and the logistical requirements of individual housing and feeding, in accordance with previous comparable dairy cow nutrition studies.

Each group was fed a different concentrate feed composed of SBM and FPS (Table 1). The control group was fed a concentrate feed containing SBM (120 g/kg) formulated to provide 185 g CP/kg dry matter (DM) and a metabolizable energy (ME) content of 2600 kcal/kg. This feed is widely regarded as the optimal choice in the region where the farm is located. The three experimental groups were fed the following diets, in which SBM was replaced with varying amounts of FPS. The FPS30 group was fed 30 g FPS/kg and 75 g SBM/kg; the FPS40 group, 40 g FPS/kg and 60 g SBM/kg; and the FPS50 group, 50 g FPS/kg and 45 g SBM/kg.

Before the study, the cows’ DM intake (DMI) consisted of a mixture of wheat straw, oat straw, corn stover, and wheat products, totaling approximately 8 kg/day. The DMI target during the trial was 12 kg/day, with 50% of the DM derived from concentrate feed and 50% from forages. Consequently, the selected cows underwent a 21-day pre-trial adaptation period to adjust to the dietary modification.

The chemical composition of the FPS is shown in Table 2. It was given to cows as part of their daily total mixed ration (TMR, Table 3) via the experimental feeds (Table 1) for 45 days following the 21-day adaptation period (total study duration = 66 days). Throughout the trial, the DMI was determined and recorded by weighing the feed remaining in the feed trough and calculating its DM content daily. Feed conversion ratio (FCR) was calculated as total DMI divided by 4% fat-corrected milk (FCM) yield, which was calculated with the following formula: 0.4 × MY (kg) + 15 × milk fat yield (kg).

### 2.2. Milk and Blood Sampling

The cows were milked twice daily (at 06:00 and 18:00) using a portable milking machine that operated at a vacuum of 44 kPa (model no. PLS-2/1; Sezer, Bursa, Turkey). MY was determined by transferring the milk contents of the portable milking machine to an empty, weighed container after milking, then weighing the container and recording the value. Final MY (FMY) was determined from the 72 MY observations obtained during the last 3 days of the study period.

To assess the milk’s chemical composition (DM, fat, solids-non-fat [SNF], protein, lactose, and mineral content), density (mg/mL), and freezing point (FP, °C), 100 mL milk samples were collected in plastic milk tubes from each cow during the last 3 days of the trial and sent for testing at the Department of Animal Science, Faculty of Agriculture, Ondokuz Mayis University, in a cold chain bag (+4 °C) on the same day. The milk samples were analyzed using a LactoStar device (Funke-Gerber, Berlin, Germany) equipped with a conductometric sensor. At least three replicates were performed per sample. The fat-to-protein ratio (FPR) was calculated as the fat percentage divided by the protein percentage.

Blood samples were collected twice from the cows. The first sample was collected on the day of selection, and the second sample was collected at the end of the trial (on day 66). Approximately 20 mL of blood was collected from the vena jugularis using a sterile needle and syringe and then transferred to serum separator tubes. Next, the tubes were centrifuged at 3000× *g* and 4 °C for 15 min to separate the serum. Then, the serum levels of glucose, triglyceride, blood urea nitrogen (BUN), calcium (Ca), phosphorus (P), and magnesium (Mg) were measured using an autoanalyzer (Cobas Integra 400 Plus; Roche Diagnostics, Mannheim, Germany). Aspartate aminotransferase (AST) and alanine aminotransferase (ALT) enzyme activities were determined using an autoanalyzer (A25; Biosystems S.A., Barcelona, Spain) and the UV-kinetic method (International Federation of Clinical Chemistry, Milan, Italy).

### 2.3. Statistical Analysis

All statistical analyses were performed using SPSS Statistics (version 25.0; IBM Corp., Armonk, NY, USA), and *p*-values < 0.05 were considered statistically significant. Continuous variables were assessed for normality and homogeneity of variances using Shapiro–Wilk and Levene’s tests, respectively. Variables expressed as percentages were arcsine-transformed before analysis to normalize the distribution of residuals; however, the actual percentages are reported. The experimental data was analyzed as a randomized complete block design, with each cow as the experimental unit. The statistical model included treatment (four groups) as the main fixed effect and parity (2nd, 3rd, and 4th lactation) as the blocking factor to account for variation associated with parity. Statistically significant differences among treatments were determined by Tukey-adjusted pairwise comparison. Whilst the MY and milk composition values reflect the average measurements taken over the last three consecutive days of the experiment, the blood biochemistry parameters were determined solely from samples taken on the final day. Consequently, each cow provided a single observation for each response variable. Correlations between FPS intake and MY, fat content, SNF, protein content, lactose content, density, FP, mineral content, DM, and FPR were examined using linear regression.

## 3. Results

The pre-trial milk samples had average DM, fat, SNF, protein, lactose, and mineral contents of 11.16%, 3.17%, 7.99%, 3.53%, 3.63% and 0.80%, respectively (Table 4). The average IMY across the 24 cows was 11.60 ± 0.94 kg (Table 5). All pre-trial data in the study are presented descriptively regarding the animals employed.

At the end of the trial (day 66), DMI, milk protein content, and milk density did not differ significantly across groups (Table 5). However, FMYs were significantly higher in the FPS50 group than in the Control, FPS30, and FPS40 groups (*p* < 0.05). In addition, milk fat, DM, SNF, and lactose contents were significantly higher in the FPS groups than in the Control group (*p* < 0.05). Moreover, milk mineral content was significantly higher in the Control and FPS50 groups (*p* < 0.05). Regarding milk physical properties, FP was significantly lower in the FPS30 and FPS40 groups (*p* < 0.05).

Serum biochemistry parameters did not differ significantly across groups on day 0 (Table 6). At the end of the trial (day 66), serum glucose, triglycerides, AST, and ALT did not differ significantly across groups. However, BUN, Ca, P, and Mg contents differed significantly between the FPS and Control groups (*p* < 0.05). BUN ranged from 5.42 to 7.21 mmol/L across groups and was highest in the Control group and lowest in the FPS40 group. Thus, diets formulated by substituting SBM with FPS resulted in lower BUN levels. Moreover, serum Ca and P concentrations ranged from 1.60 to 1.88 mmol/L and 1.67 to 2.23 mmol/L across groups, respectively, and were lowest in the FPS50 group, while serum Mg concentrations ranged from 0.58 to 0.80 mmol/L across groups and were lowest in the FPS40 group. Thus, diets formulated by substituting SBM with FPS resulted in lower serum mineral content.

Correlations between FPS intake and milk quantity/quality traits were examined (Table 7). FPS intake correlated positively with MY (*R* = 0.680), fat content (*R* = 0.665), SNF content (*R* = 0.566), lactose content (*R* = 0.653), DM content (*R* = 0.706), and FPR (*R* = 0.507), and negatively with milk density (*R* = −0.429) and mineral content (*R* = −0.271).

## 4. Discussion

This study examined the effects of substituting varying proportions of SBM with FPS containing NPN (Based on the total amino acid content of FPS, containing 635 g CP/kg DM, amino acid to crude protein ratio is 51.03% [Table 2]) in feed on milk production, with all diets providing similar starch, neutral detergent fiber (NDF), acid detergent fiber (ADF), CP, and ME contents. The findings showed significant differences across groups in MY, milk quality, and blood parameters. They are consistent with previous studies reporting increased MY when a portion of true protein is substituted with NPN [21,22]. Moreover, our findings regarding MY and FCR are consistent with numerous studies that reported improved MY and FCR when dairy cattle were fed diets containing fermented feeds [23,24,25,26]. Like the present study, most prior studies used FPS produced by the fermentation of yeast industry by-products.

The increased MY and improved FCR may reflect a shift in rumen microbes toward cellulolytic fungi/bacteria, increased volatile fatty acids (VFAs), better N conversion, and microbial protein (MCP) synthesis [26,27]. The improvements in MY and FCR with fermented feed can be explained by improved protein and fiber digestion, higher rumen propionic acid content, and beneficial effects on the gut. However, in the context of our study, it is important to consider the contribution of FPS’s NPN content rather than attributing the improvements in MY and FCR solely to the fermented feed. Moreover, our study involved crossbred cows with an extremely poor nutritional history. Unlike in our study, fermented feed showed a limited but positive impact on MY and FCR in a meta-analysis [28]. Consequently, improvements in MY and FCR reflect the nutrient content of the fermented feed and the amount included in the diet, rather than the fermented feed itself.

At the end of the trial (day 66), DMI did not differ significantly across groups, consistent with prior studies that used feedstuffs with origins similar to those of the FPS used in our study [27,29,30]. One original aspect of our study is its use of vinasse, a yeast industry by-product, as the basis for fermented feed, addressing the limited data on its use in dairy cattle diets. However, studies in non-adult ruminants have reported that elevated levels of vinasse may negatively affect DMI [18,31].

Previous studies have reported that dietary starch, direct-fed microbial supplementation, dietary fiber content, forage NDF, dietary fermented feed, and dietary NPN content positively impact lactation performance, milk composition (including DM), FCR, and nutrient digestion in dairy cattle [32,33]. However, differences in milk quantity and quality among groups in our study should be attributed to the use of FPS. The content of the FPS used in place of SBM, which was produced by fermentation of vinasse containing *A. oryzae* and *B. subtilis*, yeast metabolites, inactive yeast, and wheat bran, may have affected MY and milk DM, fat, SNF, and lactose contents.

Kand and Dickhoefer [34] reported that adequate rumen ammonia concentration and balanced rumen nitrogen (N) are critical for MCP synthesis. However, MCP synthesis requires synchronous supply of fermentable energy and N [35]. MCP and rumen undegradable protein (RUP) are sources of metabolizable protein (MP) that are broken down in the small intestine and absorbed into the blood as amino acids [36]. Thus, the observed differences in MY and milk composition of crossbred dairy cattle fed diets with the same starch and fiber content but differing in FPS content can be explained by MP [37,38]. Indeed, higher MP was found to increase milk lactose content in dairy cattle [39], as it increases the flow of amino acids (especially α-lactalbumin precursors) to the mammary gland, thereby supporting lactose synthesis and higher milk volume, with lactose content remaining tightly regulated to maintain milk osmolarity [38,39].

BUN reflects the level of urea in the blood and serves as an index of dietary protein and energy status in cattle. A BUN level above 6.43 mmol/L is considered high [40,41] and indicates overfeeding of protein. In our study, BUN levels decreased with FPS intake in crossbred dairy cattle, indicating that the protein and energy content of the FPS-containing diet was better balanced for the rumen. The inclusion of fermented feed in the diet creates a favorable ruminal environment that stimulates MCP synthesis, thereby reducing milk or ruminal NH_3_-N and BUN levels [42]. However, some studies have reported that dietary utilization of NPN increases rumen ammonia levels and, in turn, BUN levels more than the utilization of true protein sources [43,44]. Nevertheless, diets that provide a balanced intake of protein types and ensure an adequate energy supply can optimize nitrogen utilization and prevent BUN levels from becoming excessively high [45]. Thus, our findings regarding BUN suggest that substituting SBM with FPS containing NPN in dairy cattle diets does not disrupt the balance of dietary protein types at the levels examined in our study. Since the examined diets provided sufficient energy for MCP synthesis, FPS can be used in dairy cattle diets due to its positive effects on ruminal fermentation.

The findings regarding serum Ca, P, and Mg levels indicate that the use of FPS increased MY and decreased serum mineral concentrations. However, some studies have reported a positive link, showing that cows with higher MYs also had higher serum mineral concentrations [46]. For example, one reported that cows with higher MYs had 6% higher serum Ca levels, 14% higher Mg levels, and 71% higher P levels [46]. The lower serum Ca and P concentrations observed in cows with higher MYs in our study may reflect increased excretion via milk [47]. In our study, because only the total mineral content of milk was determined, its specific Ca and P contents are unknown, making it difficult to draw conclusions. Furthermore, if MY, milk fat, diet, or another factor led to a decrease in the serum level of one mineral, the serum levels of other minerals may have also changed concurrently as part of overall metabolic adaptation. However, high MY significantly reduces a cow’s mineral reserves, which can lead to a temporary decrease in blood mineral levels if the cow is unable to absorb or mobilize enough minerals to meet demand. In our study, because the examined cows had a poor nutritional history, whether their body reserves were adequate to support mineral mobilization should also be considered when interpreting our findings.

Cow’s milk generally has a total mineral content of approximately 8–9 g/L, with typical Ca, P, K, Na, and Mg concentrations of 1.0–1.2, 0.9–1.1, 1.2–1.7, 0.3–0.6, and 0.09–0.15 g/kg, respectively [48,49]. Our findings showed decreased total mineral content in the milk of the FPS30 and FPS40 groups, which can be explained by the differing K content, a major mineral constituent of milk, in FPS (0.95%) and SBM (2.49%). Nevertheless, the high milk mineral content in the FPS50 group may reflect variation (0.74–0.82%) that existed before the trial due to differences in genotype and other factors (Table 4).

This study had several limitations that should be acknowledged. Firstly, although it was designed according to established scientific research methods, and efforts were made to homogenize the groups, the sample size was small, and a larger sample could have yielded clearer results. Secondly, due to limited resources, VFA production and turnover, nutrient digestion, 24 h variation in rumen pH, MCP synthesis, assessment of rumen-degradable protein (RDP) and RUP, and analysis of some blood parameters could not be performed. Nevertheless, our findings lay the foundation for further research on this topic.

## 5. Conclusions

The results of the study indicate that FPS with a high CP content obtained by fermenting vinasse positively affected MY and milk quality without adversely affecting DMI when included at 50 g/kg in place of SBM, despite being rich in NPN. Notably, it had no adverse effect on blood biochemistry under the examined conditions and reduced BUN levels. Although substituting SBM with FPS led to economically significant changes, including in MY and milk DM and fat content, further studies are required to determine its impact on milk and serum mineral composition. Further in-depth studies are needed to investigate the effects of FPS use in high-yield dairy nutrition on rumen parameters, including VFA content, gut microbiome composition, rumen pH, and MCP synthesis.

## Figures and Tables

**Table 1:** Ingredients and chemical composition of experimental concentrate diets.

Ingredients		Quantity (g/kg)			
		CC	CFPS30	CFPS40	CFPS50
Barley		245	255	255	260
Wheat		54	54	59	59
Corn		170	170	170	170
Cracked wheat		45	50	50	50
Wheat bran		190	190	190	190
Soybean meal		120	75	60	45
Sunflower meal		120	120	120	120
Fermented protein feed		-	30	40	50
Limestone		15	15	15	15
NaCl		10	10	10	10
Vit-Min Premix		1	1	1	1
Molasses		30	30	30	30
		* Chemical composition			
Dry matter	%	88.8	89.0	89.0	89.1
Crude protein		18.5	18.5	18.5	18.5
NDF		21.22	21.41	21.46	21.53
ADF		9.68	9.63	9.60	9.60
EE		2.25	2.29	2.32	2.33
Ash		5.84	5.94	6.03	6.07
Starch		35.87	36.77	37.10	37.42
ME, kcal/kg		2600	2600	2600	2600

Note: ADF, acid detergent fiber; CC, control concentrate feed; CFPS: Concentrate feed containing FPS; CP, crude protein; DM, dry matter; EE, ether extract; ME, metabolizable energy; NDF, neutral detergent fiber; SBM, soybean meal. * All chemical composition values are expressed as the percentage of DM unless otherwise noted.

**Table 2:** Chemical composition of the FPS and SBM.

Item		FPS	SBM
DM	%	96.00	89.01
CP		63.50	45.40
EE		1.30	2.51
Crude fiber		4.27	4.41
Ash		10.21	7.29
Starch		5.51	1.67
ADF		5.98	7.78
NDF		14.61	11.66
Lignin		2.71	1.18
Ca		1.70	0.41
P		0.43	0.75
Na		0.31	0.02
K		0.95	2.49
Cl		0.59	0.07
Total amino acids		32.41	42.40
Amino acid to CP ratio, % of CP		51.03	93.43

Note: All nutrient composition values are expressed as the percentage of DM unless otherwise noted. ADF, acid detergent fiber; Ca, calcium; Cl, chlorine; CP, crude protein; DM, dry matter; FPS, fermented protein source; K, potassium; Na, sodium; NDF, neutral detergent fiber; P, phosphorus; SBM, soybean meal. The amino acid composition of the FPS and SBM was determined via liquid chromatography after performic acid oxidation and acid hydrolysis (AOAC 994.12).

**Table 3:** The TMR of the control and experimental diets and their nutrient composition.

		Experimental Diets			
Ingredients (kg)		Control	FPS30	FPS40	FPS50
CC		6.74			
CFPS30			6.74		
CFPS40				6.74	
CFPS50					6.74
Corn silage, 31.4% DM		9.58	9.58	9.58	9.58
Wheat straw		0.7	0.7	0.7	0.7
Oat hay mid-maturity		1.3	1.3	1.3	1.3
Alfalfa hay		1.4	1.4	1.4	1.4
		Nutrient composition			
DM		12,009	12,022	12,022	12,029
CP	g/day/cow	1694	1696	1696	1697
NDF		4290	4304	4307	4313
ADF		2571	2570	2568	2568
Starch		3140	3200	3219	3240
CP	% of DM	14.10	14.10	14.10	14.10
NDF		35.72	35.80	35.82	35.85
ADF		21.40	21.37	21.36	21.34
Starch		26.14	26.61	26.77	26.93

Note: ADF, acid detergent fiber; CP, crude protein; CC, control concentrate feed; CFPS, concentrate feed containing FPS; DM, dry matter; FPS, fermented protein source; NDF, neutral detergent fiber.

**Table 4:** Pre-trial (day 0) qualitative milk parameters.

Milk Components ^1^		Minimum	Maximum	Mean
Fat		3.00	3.33	3.17
DM		10.79	11.69	11.16
SNF	%	7.57	8.38	7.99
Protein		3.23	3.66	3.53
Lactose		3.41	3.85	3.61
Mineral		0.74	0.82	0.80
Milk physical traits ^1^				
Density (mg/mL)		1.02	1.03	1.02
FP (°C)		−0.60	−0.49	−0.54

Note: DM, dry matter; FP, freezing point; SNF, solids-non-fat. ^1^: The data presented herein derives from 24 milk samples obtained on the first day of the trial. Pre-trial measurements are presented solely to describe the initial characteristics and comparability of experimental groups. These variables were not considered response variables in the statistical analyses.

**Table 5:** Effects of different protein sources on performance and milk quantitative-qualitative parameters of crossbred dairy cattle.

Item		Control	FPS30	FPS40	FPS50	SEM	*p* Value
^1^ IMY, kg		11.60 ± 0.94					
^2^ FMY, kg		14.80 ^c^	16.56 ^b^	16.80 ^b^	18.20 ^a^	0.545	0.043
DMI, kg/day		11.84	11.75	11.79	11.76	0.014	0.669
FCR		0.91 ^a^	0.75 ^ab^	0.71 ^ab^	0.64 ^b^	0.011	0.018
^3^ Milk components							
Fat		3.19 ^b^	3.64 ^ab^	3.93 ^ab^	4.07 ^a^	0.113	0.021
DM		11.62 ^b^	13.32 ^ab^	13.31 ^ab^	13.79 ^a^	0.261	0.013
SNF	%	8.43 ^b^	9.67 ^a^	9.37 ^ab^	9.71 ^a^	0.190	0.047
Protein		3.36	3.62	3.46	3.60	0.070	0.567
Lactose		4.41 ^b^	5.34 ^a^	5.15 ^a^	5.28 ^a^	0.116	0.007
Mineral		0.82 ^a^	0.70 ^b^	0.69 ^b^	0.81 ^a^	0.018	0.001
^3^ Milk physical traits							
Density (mg/mL)		1.04	1.03	1.03	1.03	0.001	0.237
FP (°C)		−0.60 ^a^	−0.51 ^b^	−0.52 ^b^	−0.55 ^ab^	0.011	0.011

Note: DM, dry matter; DMI, dry matter intake; IMY, initial milk yield; FCR, feed conversion ratio; FMY, final milk yield; FP, freezing point; SEM, standard error of the mean; SNF, solids non-fat. ^a,b^ Means within a row that do not share the same superscript differ significantly (*p* < 0.05). ^1^ The presented data represent the daily MYs of all selected cows before the trial. ^2^ The presented data derive from 72 MY observations obtained during the last 3 days of the trial. ^3^ The presented data derive from 72 milk sample observations obtained during the last 3 days of the trial. Pre-trial measurements are presented solely to describe the initial characteristics and comparability of experimental groups. These variables were not considered response variables in the statistical analyses.

**Table 6:** Pre- and post-trial blood biochemistry of cows employed in the experiment.

Item	CON	30FPS	40FPS	50FPS	SEM	*p* Value
	* Pre-Trial (Day 0)					
Serum parameters, mmol/L						
Glucose	3.27	2.84	3.05	2.79	0.093	0.299
Triglyceride	0.18	0.18	0.17	0.17	0.012	0.974
Urea nitrogen	2.43	2.28	2.28	2.21	0.133	0.966
Ca	2.24	2.26	2.16	2.25	0.056	0.660
P	2.10	2.12	2.00	2.02	0.059	0.707
Mg	0.73	0.76	0.76	0.71	0.022	0.820
Enzyme activity, U/L						
ALT	23.60	26.80	23.20	27.00	1.165	0.589
AST	73.80	77.25	74.20	69.00	1.342	0.228
	Post-trial (day 66)					
^1^ Serum parameters, mmol/L						
Glucose	2.55	2.26	2.38	2.36	0.100	0.413
Triglyceride	0.11	0.13	0.17	0.13	0.013	0.467
Urea nitrogen	7.21 ^a^	6.49 ^ab^	5.42 ^b^	5.80 ^b^	0.262	0.044
Ca	1.88 ^a^	1.77 ^a^	1.72 ^a^	1.60 ^b^	0.055	0.041
P	2.23 ^a^	1.85 ^ab^	1.91 ^ab^	1.67 ^b^	0.089	0.039
Mg	0.80 ^a^	0.65 ^b^	0.58 ^b^	0.67 ^b^	0.028	0.041
Enzyme activity, U/L						
ALT	24.50	22.20	19.00	23.20	1.515	0.730
AST	67.25	69.00	59.00	62.60	2.888	0.564

^a,b^ Means within a row that do not share the same superscript differ significantly (*p* < 0.05). * Pre-trial measurements are presented solely to describe the initial characteristics and comparability of experimental groups. These variables were not considered response variables in the statistical analyses. ^1^ The data presented herein derives from 24 milk samples obtained at the end of the trial.

**Table 7:** Regression coefficients between FPS intake and milk components in cows fed with feeds containing different levels of FPS, parameters of the equations, and correlation coefficients.

Variable		α	b	ε	t	R
Independent	Dependent					
FPS	MY	3.173	0.018	0.005	3.932 **	0.680
	Fat	2.977	0.001	0.078	3.779 **	0.665
	SNF	8.548	0.025	0.009	2.912 **	0.566
	Protein	3.394	0.004	0.004	1.085	0.248
	Lactose	4.515	0.018	0.005	3.661 **	0.653
	Density	1.040	<0.001	<0.001	−2.015	−0.429
	FP	−0.586	0.001	<0.001	2.221 *	0.464
	Mineral	0.793	−0.001	<0.001	−1.194	−0.271
	DM	11.721	0.043	0.010	4.231 **	0.706
	FPR	0.943	0.004	0.002	2.494 *	0.507

Note: α, constant; b, regression coefficient; ε, standard error of the regression coefficient; t, t-statistics; R, correlation coefficient; DM, dry matter; FP, freezing point; FPR: fat-to-protein ratio; MY, milk yield; SNF, solids-non-fat. Significance: *, *p* < 0.05; **, *p* < 0.01.

## Data Availability

The raw data supporting the conclusions of this article will be made available by the authors on request.

## Abbreviations

The following abbreviations are used in this manuscript:

ADF	Acid detergent fiber
ALT	Alanine aminotransferase
AST	Aspartate aminotransferase
BCS	Body condition score
BUN	Blood urea nitrogen
BW	Body weight
CP	Crude protein
DIM	Days in milk
DM	Dry matter
DMI	Dry matter intake
EE	Ether extract
FCM	Fat-corrected milk
FCR	Feed conversion ratio
FMY	Final milk yield
FP	Freezing point
FPS	Fermented protein source
IMY	Initial milk yield
MCP	Microbial protein
ME	Metabolizable energy
MP	Metabolizable protein
MY	Milk yield
NDF	Neutral detergent fiber
NPN	Non-protein nitrogen
RUP	Rumen undegradable protein
SBM	Soybean meal
SEM	Standard error of the mean
SNF	Solids-non-fat
TMR	Total mixed ration
VFA	Volatile fatty acid
